# Ovarian Tissue Cryopreservation in Children and Adolescents

**DOI:** 10.3390/children9081256

**Published:** 2022-08-19

**Authors:** Angeliki Arapaki, Panagiotis Christopoulos, Emmanouil Kalampokas, Olga Triantafyllidou, Alkis Matsas, Nikolaos F. Vlahos

**Affiliations:** Second Department of Obstetrics and Gynecology, “Aretaieion” Hospital, Faculty of Medicine, National and Kapodistrian University of Athens, 11528 Athens, Greece

**Keywords:** ovarian, tissue, cryopreservation, children, adolescents, fertility, preservation, young, cancer, gonadotoxic

## Abstract

Cancer during childhood and adolescence remains a major public health issue, affecting a significant portion of this age group. Although newer anti-cancer treatments have improved survival rates, this comes at a cost in terms of gonadotoxic effects. As a result, the preservation of fertility is important. Ovarian tissue cryopreservation, one of the newest methods, has some advantages, especially for prepubertal patients: no need for ovarian stimulation, thus, no further risk for estrogen-sensitive cancer types, and preservation of more and better-quality primordial follicles of the ovarian cortex. The most frequent indications include treatment with alkylating agents, ovarian-focused radiotherapy, leukemias, lymphomas, brain and neurological tumors, as well as Turner syndrome and benign hemoglobinopathies. An expected survival exceeding 5 years, the absence of systematic disease and an overall risk of premature ovarian insufficiency over 50% are among the criteria that need to be fulfilled in order for a patient to undertake this method. Orthotopic transplantation is more frequently used, since it can allow both live birth and the recovery of endocrine function. Reimplantation of malignant cells is always a major risk and should always be taken into consideration. Histological analysis, as well as immunohistochemical and molecular methods, are needed in order to improve the search for malignant cells before transplantation. Ovarian tissue cryopreservation appears to be a method with specific benefits, indications and risks which can be an important tool in terms of preserving fertility in younger women.

## 1. Introduction

Despite the fact that pediatric cancer treatment has demonstrated major advances during the last few years, leading to an important decline in subsequent mortality (especially in North America and Western Europe [1]), cancer is still a major public health issue, since it is one of the most prevalent causes of death in the world and the second most prevalent cause in developed countries [2].

In the United States, cancer is—following accidents—the most frequent cause of death in children. Approximately 10,500 children (ages 0 to 14 years) and 5500 adolescents (ages 15–19 years) will be diagnosed as new cancer cases, while 1050 children under the age of 15 are expected to die from cancer in 2022. The most frequent types of childhood–adolescence cancers are leukemia, brain cancer and other nervous system tumors and lymphomas [3].

The drastic overall increase in survival rates among cancer patients seems to be correlated with the use of newer, more effective treatments in higher-income countries [4]. Pediatric cancer treatment cure rates have been improved, even up to 90% in certain types of the disease [5]. As a result, the population of childhood cancer survivors has also increased. It is estimated that 1 in 530 adults aged between 20 and 39 years old has survived cancer during childhood [6].

Apart from the direct or indirect effect of specific types of cancer on the reproductive system (ovarian, breast, uterine cancer), the aforementioned survival rates come at a cost. Cancer treatments, such as chemotherapy or radiotherapy, have often gonadotoxic effects leading to ovarian failure and infertility [7,8]. Decrease in steroidogenesis as a result of ovarian failure may lead to increased odds of associated diseases, such as hypertension, cardiovascular disease, osteoporosis and menopausal symptoms, as well as the degradation of quality of life and psychological problems and distress [9,10].

The preservation of reproductive ability is of great importance. This may be achieved by various methods, such as cryopreservation of oocytes, embryos and ovarian tissue. The latter is the method followed for prepubertal girls under specific indications [11]. The aim of this study is to describe and summarize recent advances regarding ovarian tissue cryopreservation in children.

## 2. Cancer Treatment Effect on Reproductive System

Although survival rates of patients with cancer have been significantly improved due to the introduction of newer and more advanced chemotherapy and radiotherapy treatment options, ovarian damage is an unfortunate major complication [12].

Ovaries are vulnerable to cytotoxic chemotherapy agents, which act progressively and with irreversible effects [13,14,15]. Chemotherapy drugs cause apoptosis of the primordial follicles, which appears simultaneously with a decrease in levels of anti-Müllerian hormone, activating the follicles which were still intact, finally leading to an exhaustion (burn-out) of the ovarian content [15]. The final effects of chemotherapeutic drugs on reproductive ability depend on the dose, duration of chemotherapy, drug type and age during treatment [16,17]. The chemotherapy drugs are classified into low, intermediate and high-risk groups [12,15] (Table 1). The most commonly used alkylating drugs are cyclophosphamide, busulfan and procarbazine, which are rather gonadotoxic [18,19]. As their effect is independent of cell cycle stage, it may be correlated with higher odds of primordial follicle death, at least compared to other chemotherapy agents, such as plant alkaloids and platinum-based drugs [20]. The highly toxic effect of heavy metals is also reported [9].

Radiation as cancer treatment has devastating effects, especially on the primordial follicle, leading to early ovarian failure and menopause, decreased hormone production and uterine abnormalities [21,22]. When the pelvis or abdomen are within irradiation field limits, follicular damage may appear when the radiation dose reaches ≥10 Gy in post- and ≥15 in pre-pubertal girls [23,24]. Total dose, irradiation field, age at treatment and fractionation schedule play a significant role in the final risk [12,25]. If the hypothalamic–pituitary axis is affected by radiation, gonadotropic function and puberty timing may be affected, leading to oligomenorrhea and/or amenorrhea [26]. Since age is related to the number of oocyte reserves, radiotherapy in young girls leads to the appearance of premature menopause later in their life [27]. In addition, fertility may be affected from the direct effect of radiation therapy on the uterus, in terms of reducing uterine volume and damaging the structure of the endometrium and myometrium, as well as causing arrested growth in younger patients and reducing the odds of successful embryo implantation [22,26].

According to the Children’s Oncology Group (COG) Long-Term Follow-up Guidelines, alkylating agent doses that cause gonadal dysfunction show individual variation. Higher cumulative doses of alkylators or combinations of alkylators, in combination with radiation to the abdomen/pelvis, lumbar or sacral spine (from ovarian scatter) or brain/cranium (neuroendocrine axis), and any alkylators combined with pelvic radiation or total body irradiation, need to be considered regarding increased risk for ovarian hormone deficiencies and infertility [28].

## 3. Ovarian Tissue Cryopreservation

According to the guidelines, patients must be informed about the probability of infertility after anti-cancer chemotherapy and radiotherapy treatment. In addition, they must also be informed about the appropriate methods of fertility preservation [29,30].

The preservation of reproductive potential is possible through cryopreservation of oocytes, embryos or ovarian tissue. There are indications for each method, depending on age, time to initiate anti-cancer treatment and risk of early ovarian insufficiency [11,31]. Given the need for immediate initiation of anti-cancer treatment in prepubertal girls, ovarian tissue cryopreservation seems to be the best selection in order to save fertility, but not without specific indications that are further described in a following section of the paper [11,32,33]. Prior ovarian stimulation is not needed with ovarian tissue cryopreservation. This gives the opportunity for immediate administration of anti-cancer treatment and thereby is the only option for prepubertal girls with cancer, since the potential risk of stimulation of estrogen-sensitive cancer with ovarian stimulation is avoided [5,19,30,34,35,36].

The cryopreservation of ovarian tissue has the advantage of better resistance of primordial follicles of the ovarian cortex to cryoinjury, compared to mature oocytes [5]. Another advantage is the larger number of primordial follicles that are cryopreserved, leading to increased possibility of fertility for all of the graft’s lifetime, reducing the possibility of multiple IVF attempts in order to obtain pregnancy [5]. In addition, compared to embryo and oocyte freezing methods, cryopreservation offers longer preservation of fertility and protects against earlier onset of menopause [37].

Ovarian tissue cryopreservation and subsequent transplantation are something relatively new in modern medicine. However, it has been more than 65 years since 1954, when the whole process of removal, preservation and implantation was described in rats [38,39]. It was not until 1999 that Dr. Oktay performed the first ovarian transplantation with ovarian tissue that was previously preserved [40]. Later, in 2004, the first live human birth coming from ovarian tissue which was cryopreserved using the slow freeze technique and then transplanted was described by Donnez et al. [41]. During the last few years, there have been many documented studies reporting human pregnancies using ovarian tissue cryopreservation [42,43,44] or even using robot-assisted transplant techniques [45]. As a result of the gradually increasing frequency of studies that support the effectiveness of ovarian tissue cryopreservation, the American Society for Reproductive Medicine has acknowledged this method as safe and clinically accepted since 2019 [37].

Ovarian tissue cryopreservation involves laparoscopic removal of either the entire ovarian cortex, or strips of the tissue, before the initiation of anti-cancer treatments [46]. The tissue is rich in primordial follicles, yet the tissue should be fragmented and examined histopathologically, in order to avoid any presence of malignant cells and to assess the quality of the tissue in terms of primordial follicle density [15,47,48,49,50,51]. Afterwards, the cortical tissue is frozen in small fragments. Given that the patient has recovered, completed her anti-cancer treatment and has developed ovarian insufficiency, she is eligible for ovarian tissue transplantation. The tissue can be transplanted either orthotopically (at the anatomic site of the ovaries) or heterotopically (at another site of the body) [9]. Ovarian tissue cryopreservation and autotransplantation, if appropriately activated [9], has the advantage of offering not only the restoration of fertility but also the resumption of endocrine function and initiation of puberty [48,51,52,53,54]. More specifically, studies report high frequencies of endocrine function restoral, ranging from 93 to 95%, and reaching up to ten years of functionality [35,55]. Therefore, pregnancy and live birth are possible. It should be noted that the first case was described in 2015 by Demeestere et al. [56]. This was the first live birth after an autotransplantation of cryopreserved ovarian tissue which was removed before menarche, and the patient became pregnant spontaneously. As of 2018, 360 autotransplantations have been performed [55], while successful pregnancies reached 30% [57,58,59], and more than 130 live births after ovarian tissue cryopreservation and autotransplantation have been reported [14]. The situation seems to improve, as, according to the more recent literature, the number of live births after ovarian tissue cryopreservation exceeded 200 in 2020 [60], while the pregnancy and live birth rates reached 50 and 41%, respectively, in a series of 60 women in three clinical centers [61].

## 4. Indications for Cryopreservation and Autotransplantation in Children

It was not until 2007 that ovarian tissue cryopreservation that was performed only in girls with cancer was reported [62]. Since then, multiple series conducted in patients below the age of 18 years old with various oncological and non-oncological diseases have been published [11]. As mentioned before, ovarian tissue cryopreservation is essentially the only option of fertility preservation for girls before puberty and adolescents who will be under anti-cancer and gonadotoxic treatment, given the fact that ovarian stimulation and subsequent oocyte removal are not feasible in that age, mainly due to the invasive nature of the method, virginity and immaturity issues of the patient [63].

Indications include treatment with alkylating agents, pre-allograft and autologous hematopoietic stem cell conditioning, ovarian-focused radiotherapy and gonadectomy [63]. Ovarian tissue cryopreservation is also an option even in diseases other than neoplasms, such as Turner syndrome when fertility is at risk, or in the case of allogenic transplantation of hematopoietic stem cells and incidental gonadotoxic treatment (e.g., sickle cell disease) [63].

The indications for ovarian tissue cryopreservation in children (≤18 years old) seem to include hematological malignancies (leukemia, myelodysplastic syndromes), lymphomas, bone tumors, neurological neoplasms (neuroblastoma, central nervous system cancer), sarcomas and benign hematological diseases [11,63]. The most frequent malignancies considered as indications are leukemias, myeloproliferative or myelodysplastic diseases, neurological neoplasms and sarcoma [64], while the most common non-malignant diseases were Turner syndrome and benign hemoglobinopathies [11,63]. Although there are no general guidelines, ovarian tissue cryopreservation can be a beneficial option in young girls with metabolic diseases causing ovarian degeneration, such as galactosemia. According to a recent study, these girls maintain healthy follicles in early age, thus being candidates for cryopreservation. However, the fertility rates of a preserved tissue of such origin needs to be evaluated, making this method experimental for this group of patients [65]. The relative frequency of malignancies as indication for cryopreservation ranged from 67 to 95% [11,63].

## 5. Suitability Criteria for Ovarian Tissue Cryopreservation

The fact that no hormonal stimulation is needed and a large quantity of follicles can be preserved is a major advantage towards selecting ovarian tissue cryopreservation for fertility preservation. There are, however, some suitability criteria that should be considered when implementing this technique.

The risk of premature ovarian insufficiency is an important criterion for a patient’s suitability, since it should be over 50% [31]. This risk is estimated based on both the already remaining ovarian tissue quantity and quality and the type of anti-cancer therapy to be administered, in terms of the magnitude of toxic side-effects [31,66]. Although the literature suggests the cut-off limit of fifteen years of age as suitable for ovarian tissue cryopreservation before anti-cancer treatment [33], there are other studies describing that, in cases where ovarian tissue cryopreservation cannot take place before the initiation of anti-cancer treatment (such as acute leukemia cases), the exposure to therapy before cryopreservation does not have a harmful effect on final endpoint, i.e., live births [61,66,67], even though there is skepticism concerning specifically alkylating drugs and their effects on outcome [66,68].

Pelvic radiotherapy is also a factor that needs special consideration. It is reported that the reproductive system is highly sensitive to direct radiation >25 Gy during childhood, leading to infertility [69]. It seems that live births after pelvic radiotherapy are highly unlikely, probably due to local irradiation of the area and rejection of transplanted ovarian tissue because of the development of fibrous tissue after the radiation [67]. Although transplantation of ovarian tissue is not impossible in women who have received pelvic radiation treatment, the attributes of the latter, such as location and dose, need to be thoroughly evaluated before the whole procedure.

The age of patients at transplantation of ovarian tissue is crucial, since it has been described that age is inversely associated with successful outcomes [67,70]. It seems that 35 years of age is considered as a limit for cryopreservation techniques, since the quantity of primordial follicles is reduced as the woman gets older [71]. Finally, other suitability criteria include an expected over-5-year survival, absence of metastases and absence of contraindications for surgery [12].

## 6. Techniques of Cryopreservation

The ovarian tissue cryopreservation includes the surgical removal of either strips of ovarian tissue or the ovarian cortex as a whole [46].

The quantity of the removed tissue is related to the size of the ovaries and to the risk of premature ovarian insufficiency [31]. Although the removal of both ovaries is an option, ovarian biopsies seem to be enough in order to retain fertility [14]. In the case of young patients and of pelvic and total body radiotherapy, unilateral removal of the ovary is most commonly preferred [72].

This surgical procedure must take place before the anti-cancer treatment, which can be initiated immediately and independently of menstrual cycle. Since ovarian preparation or stimulation is not needed, it is the method of choice for girls before puberty. In addition, another gain from this technique is that after reimplantation, endocrine functionality can also be restored, besides fertility [15,48,73].

In general, the most common techniques for ovarian tissue cryopreservation include vitrification, ultra-rapid freezing and slow freezing [74]. Variables such as the age of patients and medical condition are keys for the final selection of the optimum technique [8].

Vitrification is a technique that was first reported in 1985 and is already acknowledged as a standard method for both oocyte and embryo preservation [37,75]. It includes the solidification of the cells of interest in a glass-type form without ice crystallization, made possible through the exposure of the sample to solutions containing high concentrations of cryoprotectant agents and, subsequently, to quick cooling (at cooling rates reaching 5000 °C/min) using liquid nitrogen [76]. This procedure leads to the vitrification of cells without the formation of ice and its catastrophic effect on cells during cryopreservation [8]. It should be noted that higher concentrations of cryoprotectant agents are needed in order to avoid both extra- and intra-cellular ice crystals. However, the risk of cellular toxicity is increased [77].

Ultra-rapid freezing is a technique that is based on the direct exposure of the sample containers in liquid nitrogen, using lower quantities of cryoprotectant agents than vitrification [78]. However, this method has mainly been used for oocyte and embryo freezing, rather than ovarian tissue cryopreservation [8].

The slow-freezing technique was introduced in 1966 [79]. It makes use of programmable freezers, in order to obtain controlled freezing. The freezers are programmed to a cooling rate of 1.5 °C/min, making use of both liquid nitrogen and lower concentrations of cryoprotectant agents compared to those used in vitrification [10]. The whole procedure requires many hours [80]. This technique is a rather straightforward and efficient method and is the most commonly used in ovarian tissue cryopreservation, in terms of leading to successful births [31,81]. The main advantage of this type of cryopreservation method is the lower concentration of cryoprotectant agents that is required, thus reducing the risk of tissue deformation and damage [82]. These agents are dimethyl sulfoxide (DMSO), propanediol or ethylene glycol (EG) [19]. On the other hand, ice crystal formation is a result of this method, leading to stromal cells damage. This method is long-lasting and rather expensive, at least compared to vitrification [82,83]. It should also be noted that the slow-freezing technique is by far the most commonly used method of ovarian tissue cryopreservation, although it is a time-consuming and expensive procedure [76,80]. Slow freezing is by far the most preferred technique worldwide in terms of successful ovarian follicle preservation and live birth after ovarian tissue transplantation, since there is a significant amount of published evidence that supports this technique, especially compared to vitrification, where clinical data are rather rare [84]. Specifically, 131 pregnancies and 75 live births are reported (and more than 200 are estimated as of 2020) after slow freezing and transplantation, whereas only 4 births following the vitrification technique are described [37,60,85,86]. In addition, slow freezing is also related with successful restoration of endocrine function in 1 to 20 months after transplantation (mean time: 3–5 months) [37]. Although vitrification looks promising, there is a lack of sufficient data and more research is required in order to alter the current trend of slow-freezing as the method of choice [37].

## 7. Autotransplantation Methods

Re-implantation of ovarian tissue can be achieved using two methods: orthotopic and heterotopic transplantation. Orthotopic transplantation consists of implanting ovarian tissue inside the peritoneal cavity, into the remaining ovary, ovarian fossa or into a broad peritoneal ligament [19]. Heterotopic transplantation consists of implanting ovarian tissue into locations other than the peritoneal cavity, such as the subcutaneous abdominal wall, beneath the peritoneum, the rectus muscle and the forearm [19].

Orthotopic transplantation has some advantages: as has been well documented [87], it can lead to live births even through spontaneous pregnancy. The recovery of endocrine function is observed in over 95% of cases, begins 2–9 months after grafting and holds its functionality even up to 7 years [88]. It should be noted, however, that the duration of the graft function is susceptible to an expected loss of follicles during cryopreservation, and during reimplantation due to ischemic injuries [89,90]. According to the literature, by using this technique, live birth rates reached up to 41.6% [61]. However, being a surgical technique, the rate of complications is notable, ranging from 2 to 7.1/1000 cases [91]. At this time, orthotopic transplantation is the method of choice when live birth is the goal of autotransplantation [92].

Heterotopic ovarian tissue reimplantation has specific strengths, such as: simplicity, cost-effectiveness, less invasive procedure, feasibility when pelvic adhesions are present not allowing orthotopic transplantation [12,93]. On the other hand, spontaneous pregnancy is not possible and although in vitro fertilization is an option, the experience is very limited [94,95]. In fact, heterotopic transplantation is preferred when only the recovery of natural endocrine function is the goal of treatment, mainly for its advantages [96].

Ovarian tissue cryopreservation through heterotopic transplantation has already been described [53,54], resulting in recovery of endocrine function and puberty when the tissue was cryopreserved at 10 years of age. Although the experience is still limited, it seems that rates of ovarian functionality, successful pregnancy and live births are close to the already documented rates in adult patients [63].

## 8. Risk Considerations

Ovarian tissue cryopreservation, as with any other invasive medical procedure, is not free of risks and complications. The greatest risk is the potential of reimplantation of malignant cells together with the ovaries [97]. This is a real risk, since in the majority of patients, cryopreservation is implemented before the initiation of anti-cancer treatment [15]. The risk depends on the type of cancer and their ovarian involvement. There are low-risk (<0.2%) [97,98,99] malignancies, such as early stages of breast cancer (Stage I–III), squamous cell carcinoma of the cervix, Hodgkin lymphoma and Wilms’ tumor. Moderate risk (0.2–11%) appears in cases of breast cancer stage IV, Ewing sarcoma, adenocarcinoma of the cervix and non-Hodgkin lymphoma [5,97,99]. Finally, there is high risk (over 11%) when leukemia, neuroblastoma and Burkitt lymphoma are present [63]. Especially in patients with acute leukemia, malignant cells can be traced in the blood, raising the risk of reimplanting them when transplanting the ovarian graft [100].

It is a common opinion that patients considered as high-risk cases for reimplantation of malignant cells cancers should not undergo ovarian tissue cryopreservation and subsequent transplantation, or at least the ovarian tissue should be resected after multiple cycles of chemotherapy in order to reduce the possibility of the existence of malignant cells in the ovaries [5]. Although four live births have been described in patients with ovarian tissue collected after chemotherapy for leukemia [63], the risk of damaging the ovarian tissue with chemotherapy is existent and may have a negative effect on the quality and longevity of the draft [101]. Nonetheless, a thorough histological analysis of the ovarian tissue before cryopreservation needs to always be performed before reimplantation, in order to avoid the risk of transplantation of cancer cells [102,103]. Moreover, the aforementioned type of analysis may not be enough. Even if histological analyses are performed in patients that are theoretically in total remission of disease, the risk, although low, cannot be neglected [104]. Although better methods of detection of malignant cells are still being investigated [48,50,51,105], it seems that immunohistochemical testing for disease-specific markers, fluorescence in situ hybridization, molecular analysis and 6-month observation of immunodeficient animals which have been transplanted with ovarian tissue of interest complement histological analysis in search of malignant cells [85,103,106]. Given the fact that research in the field of evaluating new methods of detection of malignant cells is rather intensive in order to reduce to a minimum or even eliminate the risk of reimplantation, ovarian tissue cryopreservation should always be proposed in patients that are eligible. For example, in patients with sarcoma, ovarian tissue cryopreservation should still be an option, since it seems that there is no particular risk of ovarian involvement in their ovarian tissue [67].

Another described complication of ovarian tissue cryopreservation is the dysfunctional folliculogenesis after autotransplantation [107,108]. This can be caused by lack of synchronization between oocyte maturation and granulosa cells, hormonal imbalance due to increase in the levels of follicle-stimulating hormones, ovarian damage and low quantity of ovarian reserve. In addition, poor revascularization leading to ischemia is also a risk factor for loss of follicles [19,107]. Ischemia may also be induced by radiotherapy directed to the pelvic region, in terms of damaging endometrium and both the structure and functionality of uterine muscle, thus altering blood flow in the pelvis [69]. This effect seems also to be dose-dependent and leads to the inability of having a successful pregnancy in those auto-transplanted after local radiotherapy [105,109].

Malignant transformation of ovarian tissue after autotransplantation is also a reported risk. Since the main reason for the whole procedure of ovarian tissue cryopreservation is cancer, it should not be neglected that these patients may have increased risk for cancer in other organs, including the ovaries. For example, patients with known BRCA-1 and BRCA-2 mutations have an increased risk, reaching up to 60% for the development of ovarian cancer [110,111]. These patients should avoid orthotopic transplantation [5].

## 9. Conclusions

In this review article, we tried to summarize the latest scientific information about ovarian tissue cryopreservation, especially in younger women. It can be an option when anti-cancer treatment is needed immediately or has already been initiated. After transplantation, endocrine function is recovered, and fertility rates are satisfying. Although this is a recently implemented method, recently reported data describing live births after autotransplantation suggest that cryopreservation can be a helpful option regarding fertility preservation. Leukemia, sarcomas and malignant neurological diseases are the types of cancer that comprise the most frequent indications for ovarian tissue cryopreservation and autotransplantation in children. Ovarian tissue cryopreservation is, of course, a complicated and invasive, yet hopeful medical procedure, that gives an opportunity to girls and young women surviving cancer to keep their fertility and ability to give birth. However, the risks of this method, mainly the possibility of reimplanting cancer cells, need to be totally eliminated in order to improve its safety. Young girls and their parents should make critical decisions about their health and fertility options, such as cryopreservation. Their emotional status, as well as the need for experienced personnel that will help them through the ordeal, should be considered [112]. Guidance about future fertility and preservation methods should not involve one physician. Under ideal conditions, a multidisciplinary team consisting of treating physicians (oncologists) and of a specialized gynecologist and endocrinologist should discuss options with the patient in order to make the informed and appropriate decision. There is always a possibility of secondary ovarian failure and subsequent endocrinal disorders affecting growth and fertility in some young girls, and this must be evaluated by a proper scientific team.

As ovarian tissue cryopreservation is not yet widely implemented, further research and discussion may make this procedure a standard of care for women who may lose their fertility due to ovarian damage caused by cancer and anti-cancer treatment. Further experience, as gained from further clinical studies with patients autotransplanted with ovarian tissue harvested from early age, will hopefully lead to newer, more robust guidelines for improving their life and fertility.

## Figures and Tables

**Table 1:** Chemotherapeutic drugs classification according to ovarian toxicity risk.

High Risk	Intermediate Risk	Low Risk
Cyclophosphamide	Cisplatin	Methotrexate
Ifosfamide	Adriamycin	5-Fluorouracil
Chlorambucil	Carboplatin	Vincristine
Melphalan	Doxorubicin	Bleomycin
Busulfan		Actinomycin D
Nitrogen mustard		Vinblastine
Procarbazine		Mercaptopurine
Nitrosoureas		

## Data Availability

Not applicable.
